# Supplementary material for: How to support a co-creative research approach in order to foster impact. The development of a Co-creation Impact Compass for healthcare researchers
Source: PLoS One. 2020 Oct 12;15(10):e0240543. doi: 10.1371/journal.pone.0240543 (PMC7549764; doi:10.1371/journal.pone.0240543)
Supplement: S1 File — (DOCX) [file pone.0240543.s001.docx]

**S1 File: Description of the two case studies**

**Case 1: Smoking cessation**PhD student X enthusiastically started his new job in a four-year project: developing an e-health intervention for practice nurses in general practices to improve their adherence to smoking cessation guidelines. The research team had described the research proposal in a detailed way to secure funding. According to this plan, X started with interviews with practice nurses to explore current counselling practices and their needs regarding web-based adherence support. Based on these results, X developed a web-based computer-tailored program and conducted usability tests with some behavior change experts and nurses. He evaluated the program’s cost-effectiveness in a randomized controlled trial. The results indicated some positive program effects. However, usage of the program by the nurses appeared to be quite limited. The research project ended with four peer-reviewed publications and a successful defense of the thesis. After the funding period of the research project, the web-based intervention was no longer available as there was no owner who wanted to invest in further development and implementation of the tool.

**Case 2: Interprofessional learning**Six months ago, PhD student N started with an action-based research study. The study aimed at building knowledge and educational support for the development of interprofessional competencies by trainees in family medicine. Step 1 includes the development of a competency profile regarding interprofessional collaboration by a family physician in daily practice. A nominal group method will be used to guarantee a bottom-up, co-creative development process with professionals in primary care. Step 2 will include a needs assessment among trainees in family medicine to develop interprofessional collaboration competencies in workplace based learning, and the needs of supervisors and allied health professionals to support their learning process. It will be studied by means of a generative research method. Based on steps 1 and 2, didactic tools will be developed, refined, and evaluated in an iterative process.
