# Supplementary material for: How to support a co-creative research approach in order to foster impact. The development of a Co-creation Impact Compass for healthcare researchers
Source: PLoS One. 2020 Oct 12;15(10):e0240543. doi: 10.1371/journal.pone.0240543 (PMC7549764; doi:10.1371/journal.pone.0240543)
Supplement: S2 File — (DOCX) [file pone.0240543.s002.docx]

**Overview of co-creative tools**

| 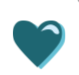 | **We want to understand our target group** |  |
| --- | --- | --- |
| Context mapping | Participants create a mood board to express their positive and negative experiences with a defined situation. It provides a deeper understanding of latent and tacit knowledge and feelings of users that can serve as a starting point for a development process. | [1] |
| Shadowing | An observational method to collect information about daily activities of individuals in their natural environment. The researcher closely ‘shadows’ someone in a predefined situation, depending on the aim of the observation. During this set period, the researcher does not interfere to avoid the participant to deviate from his/her natural behavior. | [2,3] |
| Customer journey | A visualization of a user’s experience with a service or product, outlined in a structured story, a user map. The customer (user) journey provides insight into the user’s actions, feelings and perceptions during several touchpoint in the interaction with the service or product. Based on the user map, potential barriers and opportunities for improvement can be discussed. | [4,5] |
| Day in the life | A visual representation (storyboard that include a timeline of actions and explanatory text) of one day in a life of a person. The researcher collects stories, or observes a participant in the location and context of the usual activities, and records events to understand the activities during the day from the participant’s point of view. | [6] |
| Empathy mapping | A visual map that captures users’ attitudes and behavior by asking what they think and feel, say and do, see and hear about a certain situation. The answers to these questions provides insight into the users’ pains and gains and creates a better understanding of the target group. | [7] |
| Personas | A profile of an archetypical description of end-users, describing individual behavior patterns and characteristics. Personas provide a ‘face’ to the target group and facilitates a common understanding of them. | [8] |

| 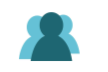 | **We want to get insight into our stakeholders** |  |
| --- | --- | --- |
| Stakeholder mapping | A visual method to identify the different stakeholders in a project and map their mutual relationships. All relevant key partners in a project are identified and structured according to their importance and influence in the project | [9-11] |
| Participation game | A tool that facilitates a discussion about the roles of the different stakeholders regarding the level of participation during the different phases of a project. | [12] |
| Value Pursuit | A visual model to structure the dialogue between stakeholders about their expectancies, attributions and challenges they face related to the shared goals of the project. The structured conversation helps to identify how stakeholders can be of value to one another, and it will encourage collaboration. | [13] |

| 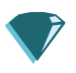 | **We want to define the value proposition** |  |
| --- | --- | --- |
| Value Proposition Canvas | A visual model to point out the pains where potential end-users struggle with, and the gains, which they strive for. The identified pains and gains are linked to the potential benefits of the new product in order to create value for the target group. Information from the tools for ‘understanding our target group’ can be used to identify the pains and gains of that group. | [14]  [15] |

| 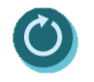 | **We want to develop, improve and test our tool/service** |  |
| --- | --- | --- |
| Prototyping | Creating a draft model or prototype for user testing. Prototypes can be cardboard scale models, paper drawings, role-plays, or a video’s. | [16] |
| Usability testing | In a think aloud test, participants verbalize their line of reasoning and offer feedback while interacting with a system or tool. In a near-live usability test, participants interact with an actor (i.e. patient, healthcare professional), a system or a tool in a simulated clinical environment. | [17]  [18] |
| Heuristic evaluation | Potential users review the usability of an interface (app, website) according to accepted usability principles. Multiple assessors, who score the product individually, fill in a comprehensive checklist of heuristics. The analysis results in a list of potential usability issues. A well-known source are the heuristics developed by Jakob Nielsen. | [19]  [20] |

| 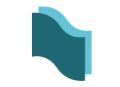 | **We want to develop a valorization plan** |  |
| --- | --- | --- |
| Business Model Canvas | A tool to discuss, define and communicate the business and valorization plan in order to create sustainable impact. The model, which includes nine fundamental elements of a business, is presented on a canvas to facilitate a group discussion about creating value (customers segments, value proposition), how to realize value (by key partners, key activities, key resources, customer relationships, channels) and about the cost structure and revenue streams. All building blocks can be filled up during the project. | [21]  [22] |

| 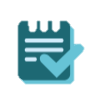 | **We want to collect information and make shared decisions** |  |
| --- | --- | --- |
| Process Mapping | A method to structure and visualize working processes by means of flow charts showing the who, what, when and how. The flow charts provide a shared focus on the systems and processes into which new interventions are introduced. | [23] |
| World café | A flexible, structured method of discussion in a relaxed environment for large groups. Participants engage in several rounds of small-group conversation. Each round is prefaced with a question. As participants carry key ideas or themes to new tables, they exchange perspectives, greatly enriching the possibility for creative new insights. | [24]  [25] |
| Thinking Hats | A structured method to reflect on a specific issue from different perspectives. By putting on a certain color hat - visually or metaphorically - the participant in the decision-making process is invited to think in a certain way. Each color represents a way of thinking that people use when they make decisions. | [26]  [27]  [28] |
| 100-dollar method, or  Cumulative Voting, or  100-Point Method | A simple, straightforward and intuitively appealing voting scheme where each stakeholder is given a constant amount of imaginary units (for example dollars) that can be used for voting in favor of the most important issues. In this way, the most valuable ideas are selected. | [29]  [30] |
| Nominal Groups Technique | A consensus method that is directed at problem solving, idea-generation, or determining priorities. It emphasizes individual creativity and group decision-making. In small groups, participants start with writing down their response to questions posed by a moderator. Once different ideas are established, they are evaluated, ranked, and agreed upon collectively. The top-ranked ideas are selected as the output of the group process. | [31]  [32] |

**References**

1. Visser FS, Stappers PJ, van der Lugt R, Sanders EBN. Contextmapping: experiences from practice. CoDesign 2005;1: 119-149.
2. McDonald S. Studying actions in context: a qualitative shadowing method for organizational research. Qualitative Research 2005;5: 455-473. [https://doi.org/10.1177/1468794105056923](https://doi.org/10.1177%2F1468794105056923)
3. Husebo SE, Olsen OE. Actual clinical leadership: a shadowing study of charge nurses and doctors on-call in the emergency department. Scand J Trauma Resusc Emerg Med 2019;27: 2.
4. Siddiqui S, Cruz I. A Cancer Patient Journey: Complete Review During Acute Treatment Phase. Health equity 2019;3: 403-408. <http://doi.org/10.1089/heq.2019.0046>.
5. Rosenbaum MS, Otalora ML, Ramírez GC. How to create a realistic customer journey map. Business Horizons 2017;60: 143-150. <https://doi.org/10.1016/j.bushor.2016.09.010>
6. del Rio Carral M. Focusing on “A Day in the Life”: An Activity-Based Method for the Qualitative Analysis of Psychological Phenomena. Qual Res Psychol 2014;11: 298-315.
7. Zuber CD, Moody L. Creativity and Innovation in Health Care: Tapping Into Organizational Enablers Through Human-Centered Design. Nurs Adm Q 2018;42: 62-75.
8. Wärnestål P, Svedberg P, Lindberg S, Nygren JM. Effects of Using Child Personas in the Development of a Digital Peer Support Service for Childhood Cancer Survivors. J Med Internet Res. 2017;19: e161-e161.
9. van Limburg M, Wentzel J, Sanderman R, van Gemert-Pijnen L. Business Modeling to Implement an eHealth Portal for Infection Control: A Reflection on Co-Creation With Stakeholders. JMIR Res Protoc 2015;4: e104.
10. Silver SA, Harel Z, McQuillan R, Weizman AV, Thomas A, Chertow GM, et al. How to Begin a Quality Improvement Project. Clin J Am Soc Nephrol 2016;11: 893-900.
11. Shirey MR. Stakeholder analysis and mapping as targeted communication strategy. J Nurs Adm 2012;42: 399-403.
12. de Wit M, Beurskens A, Piskur B, Stoffers E, Moser A. Preparing researchers for patient and public involvement in scientific research: Development of a hands-on learning approach through action research. Health Expect 2018;21: 752-763.
13. Rygh K, De Vos M, Raijmakers B, editors. Value Pursuit: creating value between stakeholders in policy development. Participatory Innovation Conference 2015; 2015; The Hague, the Netherlands.
14. Osterwalder A, Pigneur Y, Bernarda G, Smith A. *Value proposition design: how to create products and services customers want*. New Jersey: John Wiley & Sons, 2014.
15. Jones LP, Slade JL, Davenport F, Santos SLZ, Knott CL. Planning for Community Scale-Up of Project HEAL: Insights From the SPRINT Initiative. Health Promot Pract. 2019 Jan 24:1524839918824087. [https://doi.org/10.1177/1524839918824087](https://doi.org/10.1177%2F1524839918824087)
16. Roberts JP, Fisher TR, Trowbridge MJ, Bent C. A design thinking framework for healthcare management and innovation. Healthcare 2016;4: 11-14.
17. Rose, AF, Schnipper JL, Park ER, Poon EG, Li Q, Middleton B. Using qualitative studies to improve the usability of an EMR. J. Biomed. Inform 2005;38: 51-60.
18. Richardson S, Mishuris R, O'Connell A, Feldstein D, Hess R, Smith P, et al. "Think aloud" and "Near live" usability testing of two complex clinical decision support tools. Int J Med Inform. 2017;106:1-8. <https://doi:10.1016/j.ijmedinf.2017.06.003>
19. Zhang J, Johnson TR, Patel VL, Paige DL, Kubose T. Using usability heuristics to evaluate patient safety of medical devices. J Biomed Inform 2003;36: 23-30.
20. Nielsen, J., Mack, R.L. (Eds.). *Usability Inspection Methods*, John Wiley & Sons, New York, NY, 1994.
21. Osterwalder A, Pigneur Y. *Business Model Generation. A Handbook for Visionaries, Game Changers, and Challengers.* Hoboken, New Jersey.: John Wiley & Sons, Inc., 2010.
22. Milat AJ, Bauman A, Redman S. Narrative review of models and success factors for scaling up public health interventions. Implementation Sci 2015; 10: 113. <https://doi.org/10.1186/s13012-015-0301-6>.
23. Antonacci G, Reed JE, Lennox L, Barlow J. The use of process mapping in healthcare quality improvement projects. Health Serv Manage Res 2018;31: 74-84.
24. MacFarlane A, Galvin R, O'Sullivan M, McInerney C, Meagher E, Burke D, et al. Participatory methods for research prioritization in primary care: an analysis of the World Café approach in Ireland and the USA. Fam Pract 2017;34: 278-284.
25. Trenaman S, Willison M, Robinson B, Andrew M. A collaborative intervention for deprescribing: The role of stakeholder and patient engagement. Res Social Adm Pharm. 2019 Jul 10. <https://doi.org/10.1016/j.sapharm.2019.07.004>.
26. De Bono E. *Six Thinking Hats*. 1 ed. London: Penguin Books Ltd, 1996.
27. Cioffi JM. Collaborative care: Using six thinking hats for decision making. Int J Nurs Pract 2017;23: e12593.
28. Taie E, El kamel A. Six thinking hats as a creative approach in managing meetings in hospitals. J Nurs Educ Pract. 2013;3: 188-200.
29. Leffingwell D, Widrig D. *Managing software requirements: A Use Case Approach*. 2 ed. Boston: Addison-Wesley, 2003.
30. Tofan D, Galster M, Lytra I, Avgeriou P, Zdun U, Fouche M-A, et al. Empirical evaluation of a process to increase consensus in group architectural decision making. Inform Software Tech 2016;72: 31-47.
31. McMillan SS, King M, Tully MP. How to use the nominal group and Delphi techniques. Int J Clin Pharm 2016;38: 655-662.
32. Pagel C, Brown KL, McLeod I, Jepps H, Wray J, Chigaru L, et al. Selection by a panel of clinicians and family representatives of important early morbidities associated with paediatric cardiac surgery suitable for routine monitoring using the nominal group technique and a robust voting process. BMJ Open 2017;7: e014743.
