# Supplementary material for: How to support a co-creative research approach in order to foster impact. The development of a Co-creation Impact Compass for healthcare researchers
Source: PLoS One. 2020 Oct 12;15(10):e0240543. doi: 10.1371/journal.pone.0240543 (PMC7549764; doi:10.1371/journal.pone.0240543)
Supplement: Attachment — Submitted filename: Response to reviewers PONE-D-20-05698 R1.docx [file pone.0240543.s003.docx]

Subject: Revision PONE-D-20-05698. How to support a co-creative research approach in order to foster impact. The development of a Co-creation Impact Compass for healthcare researchers

Dear editor and reviewers,

We would like to thank you for the opportunity to revise and resubmit our article for publication in PLOS ONE. Moreover, we thank the reviewers for their comments, which have really helped us to improve our article. We have adjusted our manuscript according to your comments and suggestions. Our point-by-point response to the reviewers is set out below. The reviewers’ comments are given in italics. We refer to the line numbers of the manuscript with track changes.

During the review procedure of this manuscript, the layout and design of the compass was improved, therefore figure 3, which includes the Co-creation Impact Compass, has been changed.

**Reviewer #1:**
*The main purpose of this study is to highlight how the co-creation process can be a functional method for health researchers by helping them involving all the stakeholders and improving the impact of their researches. The paper is properly placed in the context of the previous literature by highlighting the potential of co-creation techniques but the lack of a reference framework for health care surveys. The study therefore aims to describe the process of creating a "compass" of co-creation tools and techniques that can be used by researchers in this field in an effective way (which tools/in which moment).*

1. *In the “introduction”, I recommend removing the word "demonstrates" from line 82 because this term refers to demonstrating the effectiveness of this tool, which is not the goal of this research, that is to DESCRIBE the creation of this tool.*
   Thank you for this remark. Indeed, our article describes the development process of the compass. However, the paper also presents the final instrument in the appendix, which can be used by researchers immediately. To highlight that the article includes both a description of the development process and a demonstration of the outcome, we have reformulated the sentence into: This article describes the bottom-up development process of an instrument and presents the final outcome, which is a compass to support co-creation in research practice. (line 86 vv). We have also reformulated the aim in the abstract (line 26-28).

*Moreover, I think that the structure of some parts (especially of methods and results) should be modified in order to ensure consistency and linearity in the reading of the paper. I will resume all the proposed changes here:*

2. *I would move the "Setting" part to the beginning of the methods for introducing the framework in which the study fits, and specify which of the categories* *this study falls into.*
   That is a good suggestion, thank you. We have moved ‘setting’ to the beginning of the methods (line 92 vv). We have reshuffled the text a bit to focus on the role of the co-creation team, who initiated the development of the compass to support the research teams, rather than focusing on the different projects in LIME (see 96-112).
   Moreover, in the description of our design, we have added the aim, which is to develop an instrument to help researchers improve their research (see line 116).
3. *I would remove the sub-paragraph "development process" and integrate it with the design. Too many sub-paragraphs continuously interrupt the reading. In this way, the design part is also linked to subsequent explanations of the various stages of the process.*
   We followed the suggestion from the reviewer. The sub-paragraph ‘development process’ has been integrated with the design. See line 135.
4. *In my opinion some information from the “results” section must be moved to the methods section (e.g. line 179 – 186; 286-290; 324-328 including the box describing the two cases) these are all details referring to the methodology and not to the results of the study), these would help the authors to better clarifies the methodology which is currently not very precise and, on the other hand, it streamlines the results.* We followed the suggestion from the reviewer. The description of the participants and the description of the two cases has been moved to the methods section and as S1 file in order to optimize the clarity and flow of the article (see line 205-206).
5. *I find the explanation of BMC unclear, which should be the central point of the paper. For example, what are the nine blocks? What do the nine blocks represent? How are they implemented in the compass?*
   Thank you for your feedback. We now have included both a figure and a description of the nine blocks of the BMC to the results section (line 318 vv) to provide more information about this framework. In the results section phase 3, our adjustments to the original framework of the BMC and the way in which the building blocks have been implemented in our Compass are explained. (See 370 vv)
6. *Line 320: personally, I would find a way to put the symbol legend inside the image. Putting the legend inside the article breaks the reading. Rather, in the text, I would put a brief explanation of what you mean by each category.*
   We have included the symbol legend into the image (figure 3). Each symbol refers to a question. We added these questions to the main text of the manuscript (line 385 vv).
7. *Discussion: look above for the use of the word “demonstrates” as concern line 45-456 since it explains what has been done in this study, I would move them before explaining the advantages (before line 438).*
   Thank you for the suggestion. We have removed the sentence to the first paragraph of the discussion section. Furthermore, we have reformulated the first sentence of the discussion to put an emphasis on both the development process and the final version of our compass. According to our adjustments regarding the reviewers’ first remark (see line 513-514).
8. *I suggest to add a “future direction” section and to merge here what you think are the future implications thanks to the use of this tool.*
   Our future directions in education and research were described in the last part of our discussion. To put more emphasis on this part, we have added a heading (“Future directions”, see line 568) to this section.
9. *In the conclusion section I suggest to highlight better what you mean with “impact”*
   In the discussion section (see line 576vv), we stated that we would like to know more about the effects of using the compass in terms of societal or economic impacts. We have added a referral to the literature, which suggests that outcomes are more likely to be accepted and provide societal value. Some references to the tools (included as supporting information S2) also show the effectiveness of specific tools on the research outcomes, for example personas and consensus methods. We have added these examples to the discussion section.

**Reviewer #2**

*Authors addressed a key topic in interdisciplinary applied research: engagement of all stakeholders and researchers in a specific field, that is, healthcare.*

1. *At the beginning of Authors' work, they stated that the aim is to present a "compass" to facilitate such engagement. However, it is not clear what this "compass" is: a tool; a method; a process of intervention? This is the first main weakness of this work: the goal and the nature of their work is not stated clearly.*
   Thank you for attending to this issue. Our ultimate goal was to support researchers regarding stakeholder engagement, in order to foster the impact of their work. Our needs assessment revealed that researchers needed an instrument to select helpful and valid co-creation tools, which we named: the ‘Co-creation Impact Compass’

To meet your feedback, we reformulated the aim of the article into: **This article describes the bottom-up development process of an instrument and presents the final outcomes, which is a compass to support co-creation in research practice**. (See also our comment to the first remark of reviewer 1, and line 87vv)

2. *A clear overview of existing methods and tools previously used to pursue an engagement in applied research is not presented. Thus, the added value of Authors' proposal does not emerge. At the same time, the weaknesses of previous approaches are not reported.*
   To our knowledge, a clear overview of existing methods for research practice does not exist. For that reason, researchers need a practical instrument to guide them in stakeholder engagement during their research. That is the rationale of our article. To meet your feedback, we elaborated on the researchers’ need in the problem statement of our introduction (see 78-84).
3. *This, in turn, affects the clarity of Authors' rational. Authors presented a synthetic overview of the "compass" in figure 1. Having a quick look at this image reveals that their proposal consists in: a. analysis of need; b. generation of requirements; c. alpha prototype; d. beta-prototype. This look as a normally-used approach of participatory design where all stakeholders are involved in generation and evaluation of ideas of prototypes. What does Author's idea adds to existing approaches?*
   The participatory design is indeed a normally-used approach. Figure 1 shows the iterative development process. We used the participatory action research design because we wanted our product to be useful for our target group (health care researchers). The outcome of this process, the Co-creation Impact compass, is presented in figure 3, and the co-creative tools are presented in the appendix (S2). Researchers can use this compass; the publication gives insight into the background and development process of this instrument.
4. *Authors relied on a business model as a theoretical framework. Fine...but why? how? Is it something replicable (I ask this because this choice stems from Authors' analysis of results and is not due to their analysis of existing solutions).*

Based on data from the interviews with the target group and a literature search, the business model came forward as a helpful underlying framework for the Co-creation Impact Compass.

5. *Authors stated that they involved different researchers in different evaluation phases. This is fine, but why?*
   The different phases of our development had different aims and therefore needed different respondents. In phase 1, ‘needs assessment’, our target group e.g. the LIME researchers, were our subject. In phase 3, ‘alpha testing’, we consulted experts and asked the LIME researchers to test prototype I. In phase 4, external researchers were involved as in ‘Beta testing’ is suggested. They tested our prototype II. In phase 5, ‘finalizing’, researchers and educators from Zuyd University of Applied Sciences were involved to use and evaluate prototype III.
   Involving different researchers, experts and educators increases the likelihood of a broad support of our compass in the field of research and education
6. *Is it just a data-driven approach?*

Besides consulting different researchers, experts and educators in the different phases, we also conducted a literature search in phase 2 to provide information about useful frameworks and tools for co-creation.

7. *To what extent can it be replicable and generalizable and useful compared to previous approaches?*
   The broad description of the participatory action research process, in which each phase is extensively described, increases the replicability of this approach.
   The criterion of generalizability has been met by our thick description of the different respondents and settings in each phase of the user-centred approach.
8. *Authors' title is "How to support a co-creative research approach in order to promote impact". However, impact is not assessed because the real object of their research is not clearly stated*.
   Thank you for your attention to this point. We agree that impact is not assessed in this study, because our aim was to describe the development process and present the co-creation impact as an outcome of this process. To alter misleading expectations, we replaced the word ‘promote’ in our title into ‘foster’ more impact. (see title)
   Moreover, we stated in the introduction (line 42-44) that co-creation can lead to outcomes that are more likely to be acceptable, valuable, and enduring than traditional research approaches, and that co-creation has been associated with significant societal impact through better implementation and utilization of products and services. We truly are aware of the fact that there is no linear association between co-creation and impact.
9. *I recommend Authors to work on their rational and avoid implicit discourse on the underpinnings of their approach. Otherwise, this would look just a mere post-hoc ad hoc analysis of their participatory design approach implementation in the healthcare field, without being a clear and useful information for other researchers or practitioners.*
   We hope that the revisions that we have made according to the reviewers comments and suggestions are sufficient to take away your doubts.
