# Supplementary material for: How to support a co-creative research approach in order to foster impact. The development of a Co-creation Impact Compass for healthcare researchers
Source: PLoS One. 2020 Oct 12;15(10):e0240543. doi: 10.1371/journal.pone.0240543 (PMC7549764; doi:10.1371/journal.pone.0240543)
Supplement: Attachment — Submitted filename: Response to reviewers PONE-D-20-05698 R2.docx [file pone.0240543.s004.docx]

Subject: Revision PONE-D-20-05698R1

How to support a co-creative research approach in order to foster impact. The development of a Co-creation Impact Compass for healthcare researchers

Dear editor and reviewers,

We are glad to hear that our manuscript does meet PLOS ONE’s publication criteria.

We would like to thank you for the invitation to respond to the points raised by reviewer 1, which helped us to further improve our manuscript.

In this letter, the reviewers’ comments are given in italics. We refer to the line numbers of the manuscript with track changes.

**Reviewer #1:**

1. *I would modify the abstract (27-33) by stating firstly how the instrument was created (i.e. by combining the business model with tools from design thinking) and only after pointing on the possibility that this tool (compassion) MIGHT help researchers to select valid co-creation tools which can help them to engage stakeholders, etc.*

Thank you for these remarks. We have reordered the abstract according to your suggestions. We have also reformulated the conclusion of the article by stating that the Co-creation Impact Compass might increase the researchers’ understanding of the value of co-creation, and provides help to engage stakeholders in all phases of a research project. See line 25-33.

2. *Linked to the first comments, given the importance that the tool might have for researchers, I wonder if it would not be appropriate to move the “supplementary material 2” directly into the article so that it can be directly explored (perhaps creating a single table) perhaps by briefly going into the description of the tools (which are a bit poor). If it were a problem with the number of images/tables I would recommend removing image 2 (maybe you can refer back to the original article since the model is now briefly explained in the text).*

Thanks for the suggestion to put more emphasis on the overview of co-creative tools, given its importance for the readers. We propose a stepped approach:

1. We added a single table to the manuscript, in which all tools of the Co-creation Impact compass are listed, according to the underlying aim/building block of the compass. See line 431-438.
2. The more detailed descriptions of the tools are provided in the additional file (S2). This additional file also provides references to scientific literature, in which the tools have been used in a research setting. In our opinion, this overview with descriptions and 32 extra references would not fit into the main manuscript.

The reviewer stated that the descriptions of the tools were a bit poor. We have critically reviewed the information about the features of the tools, and added some extra information. However, the descriptions may still feel a bit poor since the Co-creation Impact compass is an instrument to select tools instead of providing detailed information and manuals. We think that the references to scientific articles will help researchers in their selection process. Manuals and other practical information about these tools can easily be found on the internet and literature.

We hope that the revisions that we have made are sufficient for publication of our manuscript in PLOS ONE.

On behalf of all authors,

Anneke van Dijk, PhD corresponding author
